# Deletion of TXNIP Mitigates High-Fat Diet-Impaired Angiogenesis and Prevents Inflammation in a Mouse Model of Critical Limb Ischemia

**DOI:** 10.3390/antiox6030047

**Published:** 2017-06-29

**Authors:** Sally L. Elshaer, Islam N. Mohamed, Maha Coucha, Sara Altantawi, Wael Eldahshan, Megan L. Bartasi, Ahmed Y. Shanab, Renee Lorys, Azza B. El-Remessy

**Affiliations:** 1 Clinical and Experimental Therapeutics, College of Pharmacy, University of Georgia, Augusta, GA 20912, USA; selshaer@uthsc.edu (S.L.E.); islam.mohamed@emory.edu (I.N.M.); mcoucha@southuniversity.edu (M.C.); sara.m.altantawi@gmail.com (S.A.); weldahshan@augusta.edu (W.E.); mbartasis@gmail.com (M.L.B.); shanab79@gmail.com (A.Y.S.); rlorys@uga.edu (R.L.); 2 Augusta Biomedical Research Corporation, Charlie Norwood VA Medical Center, Downtown 6B-105, Augusta, GA 30912, USA

**Keywords:** high-fat diet, hind-limb ischemia, vascular endothelial growth factor, thioredoxin interacting protein, inflammation

## Abstract

Background: Previous work demonstrated that high-fat diet (HFD) triggered thioredoxin-interacting protein (TXNIP) and that silencing TXNIP prevents diabetes-impaired vascular recovery. Here, we examine the impact of genetic deletion of TXNIP on HFD-impaired vascular recovery using hind limb ischemia model. Methods: Wild type mice (WT, C57Bl/6) and TXNIP knockout mice (TKO) were fed either normal chow diet (WT-ND and TKO-ND) or 60% high-fat diet (WT-HFD and TKO-HFD). After four weeks of HFD, unilateral hind limb ischemia was performed and blood flow was measured using Laser doppler scanner at baseline and then weekly for an additional three weeks. Vascular density, nitrative stress, infiltration of CD68+ macrophages, and expression of inflammasome, vascular endothelial growth factor (VEGF), VEGF receptor-2 were examined by slot blot, Western blot and immunohistochemistry. Results: By week 8, HFD caused similar increases in weight, cholesterol and triglycerides in both WT and TKO. At week 4 and week 8, HFD significantly impaired glucose tolerance in WT and to a lesser extent in TKO. HFD significantly impaired blood flow and vascular density (CD31 labeled) in skeletal muscle of WT mice compared to ND but not in TKO. HFD and ischemia significantly induced tyrosine nitration, and systemic IL-1β and infiltration of CD68+ cells in skeletal muscle from WT but not from TKO. HFD significantly increased cleaved-caspase-1 and IL-1 β compared to ND. Under both ND, ischemia tended to increase VEGF expression and increased VEGFR2 activation in WT only but not TKO. Conclusion: Similar to prior observation in diabetes, HFD-induced obesity can compromise vascular recovery in response to ischemic insult. The mechanism involves increased TXNIP-NLRP3 (nucleotide-binding oligomerization domain-like receptor protein 3) inflammasome activation, nitrative stress and impaired VEGFR2 activation. Deletion of TXNIP restored blood flow, reduced nitrative stress and blunted inflammasome-mediated inflammation; however, it did not impact VEGF/VEGFR2 in HFD. Targeting TXNIP-NLRP3 inflammasome can provide potential therapeutic target in obesity-induced vascular complication.

## 1. Introduction

Over one-third (~80 million) of US adults are obese and at higher risk for developing diabetes and cardiovascular complications. The recent upgrade of obesity from a mere risk factor to a disease-state signifies the need to identify and understand obesity-mediated vascular changes independent from diabetes. Peripheral arterial disease—characterized by limited blood supply to organs other than the heart—affects nearly 8.5 million people in the United States [1]. Critical limb ischemia (CLI) is one of the major symptomatic clinical manifestations of peripheral arterial disease, which can lead to major amputation and annual mortality in 25–40% and 40% of patients, respectively [2]. Failure to establish revascularization eventually leads to amputation [3]. While the link between critical limb ischemia and diabetes is well documented [4], clinical reports suggest that obesity and insulin resistance without frank hyperglycemia could impair functional recovery after ischemic episodes [5,6]. Recent evidence from experimental models shows compromised functional recovery to ischemia similar to non-diabetic patients with insulin resistance [5,7]. Nevertheless, there is a gap in knowledge on the extent and mechanisms involved in microvascular dysfunction observed in a model of high-fat diet-induced (HFD) obesity.

Thioredoxin interacting protein (TXNIP), a physiological inhibitor for thioredoxin antioxidant system, could exert its detrimental effects via thioredoxin-independent mechanisms, which involve glucose uptake inhibition and direct activation of inflammation (reviewed in [8,9]). Increased reactive oxygen species enhances the dissociation of TXNIP from thioredoxin. This effect results in increasing TXNIP availability to interact with nucleotide-binding oligomerization domain-like receptor protein 3 (NLRP3) leading to inflammasome activation. Activated NLRP3 oligomerizes with the apoptosis-associated speck-like adaptor protein forming caspase-1 activating complex known as NLRP3 inflammasome [9]. Activated caspase-1 enzyme in turn cleaves premature pro-inflammatory cytokines including IL-1β and IL-18 into their mature form causing inflammation [10,11]. Our lab demonstrated that HFD resulted in increased oxidative stress, enhanced TXNIP-NLRP3 interaction and inflammatory markers expression. [12]. TXNIP is a glucose and calcium sensor so its expression can be modulated by a plethora of environmental changes [13]. Recently, we showed that exposing retinal glial Muller cells to palmitate—most abundant saturated fatty acid after high caloric intake—enhanced TXNIP expression via endoplasmic reticulum stress-mediated degradation of microRNA miR-17-5p compared to vehicle [14]. In vitro, exposure to palmitate triggered activation of TXNIP-NLRP3 inflammasome activation and release of IL-1β in primary retinal cells isolated from wild type mice but not from TXNIP-knockout mice [14]. However, the impact of TXNIP-NLRP3 inflammasome activation on HFD-impaired vascular recovery remains unexplored.

In response to ischemia, revascularization and growth of collaterals re-establish adequate blood flow. Vascular endothelial growth factor (VEGF) is a potent angiogenic factor, which is crucial for creating new blood vessels during embryonic development and after injury. VEGF exerts its angiogenic effect mainly via trans-auto-phosphorylation of various tyrosine residues in the intracellular domain in its receptor 2 (VEGFR-2) [15]. Reduced VEGF production has been linked to the impairment of ischemia-mediated angiogenesis in diabetes [16]. The interplay between thioredoxin system including thioredoxin, TXNIP and VEGF expression is well documented in the literature (reviewed in [17]). While one study showed that enhanced TXNIP expression stimulates VEGF expression [18], others showed that enhanced TXNIP can downregulate VEGF production [16,19,20]. Yet, the impact of HFD-mediated obesity on VEGF level or its receptor activation remains unknown. The objective for this study is to unravel the extent and mechanisms of compromised vascular recovery in HFD-induced obesity apart from diabetes-driven pathological changes. The study aimed to dissect the contribution of TXNIP-NLRP3 inflammasome activation as well as its possible impact on VEGF/VEGFR2 angiogenic signal in hind limb ischemic model.

## 2. Materials and Methods

**Animals:** We complied with the Association for Research in Vision and Ophthalmology statement for use of animals in ophthalmic and vision research, and Charlie Norwood VA Medical Center Animal Care and Use Committee (ACORP#15-04-080) in all animal experiments. Wild type mice (WT, C57Bl/6) and TXNIP knockout mice (TKO) were kept either on normal chow (WT-ND and TKO-ND) or 60% high-fat chow (Research Diet) (WT-HFD and TKO-HFD). After 4 weeks of HFD, unilateral hind limb ischemia was performed in all groups. Mice were anesthetized with isoflurane inhalation (2%). A unilateral incision was made over the left medial thigh. The superficial femoral artery was ligated proximal to the caudally branching deep femoral artery and proximal to the branching of tibial arteries. The portion of the artery between two ligation points was excised. The skin was then closed with interrupted silk sutures (S-N618R13, AD surgical, 6-0 Nylon suture). Subcutaneous analgesic (Buprenorphine, 1 μL/g, SC, Reckitt Benckiser Pharmaceuticals Inc., Richmond, VA, USA) was injected 20 min before surgery and thereafter for 3 days in addition to topical antibiotic (Multi antibiotic with pain relief, Walgreen, Deerfield, IL, USA). Laser Doppler (PeriScan PIM 3 System, Perimed Inc., Ardmore, PA, USA) was used to record blood flow in hind limbs after surgery and thereafter for 3 weeks using LDPIwin 3.1 software (http://www.calmira.net/). Body weight and fasting blood glucose were measured weekly to record changes of body weight and blood glucose levels throughout the study.

**Intra-peritoneal glucose tolerance test (IPGTT):** As previously described, all animal groups were fasted overnight and fasting blood glucose (FBG) levels were recorded as the baseline and then all groups were challenged with a bolus intra-peritoneal injection of an equal dose of glucose (2 grams/kg/ mouse body weight). After glucose injection, prandial blood glucose PBG levels were recorded at 20, 40, 60, 90 and 120 min. Analysis of the area under the curve (AUC) was performed to assess the response across all groups.

**Determination of plasma total cholesterol and triglycerides levels:** Both levels were determined using commercially available kits (Biovision Inc., Milpitas, CA, USA, Catalog # K613-100 for total cholesterol and Catalog #K622-100 for triglycerides) following the manufacturer’s protocol. Briefly, plasma samples were diluted into assay-buffer and reacted with reaction mix or treated with lipase enzyme followed by the reaction mix and incubated for 60 minutes (for triglycerides kit) and then color absorbance was measured at 570 nm.

**Determination of plasma IL-1β levels:** Plasma IL-1β levels were determined using IL-1β ELISA sensitive kit (R&D systems Catalog #MLB00C, Minneapolis, MN, USA). IL-1β concentrations were expressed as pg/mL as described previously by our group [12].

**Western blot analysis:** Gastrocnemius muscles were isolated and homogenized mechanically by mortar & pestle system. Muscles were placed in porcelain mortar, and grounded with porcelain pestles after the addition of liquid nitrogen. Powdered tissues were re-suspended in modified radioimmunoprecipitation assay (RIPA) buffer (Millipore, Billerica, MA, USA). Samples were separated by SDS-PAGE. Membranes were probed with the primary antibodies then re-probed with housekeeping gene to confirm equal loading (list of antibodies used in Table 1). Band intensities were measured using alphaEaseFC (Santa Clara, CA, USA) and expressed as relative optical density (OD) compared to normal diet control of WT and TKO, respectively.

**Slot blot analysis:** Skeletal muscle homogenates (20 µg) were immobilized on nitrocellulose membrane and nitrotyrosine was detected as described previously [21]. Biological samples of 4 different animals were run each as triplicates and scanned to obtain optical density.

**Immunohistochemistry:** After 4 weeks of hind limb ischemic insult, gastrocnemius muscles were sectioned and fixed in paraformaldehyde. Pilot studies to assess basal vascular density between WT and TKO were performed initially as follows: Gastronomes muscle sections were permealized then stained overnight at 4 °C with isolectin B4; biotinylated griffonia (bandeiraea) simplicifolia lectin I (GSL I, BSL I), (Vector Labs, Burlingame, CA, USA; 1% in 5% normal goat serum in 0.3% Triton X-PBS) followed by incubation with secondary antibody; Texas red^®^ avidin D (Vector labs, Burlingame, CA, USA; 0.5% in 5% normal goat serum in 0.3% Triton X-PBS). Percentage vascular density was calculated on isolectin-stained muscle sections by averaging number of branches across the 5 longest bathes using FIJI densitometry software version analysis (data presented in supplementary Figure S1). Vascular endothelial cell density was measured by CD31 immunostaining (1:200, Cat# 550274, BD-Biosciences). In another set of muscles, sections were incubated overnight with anti-CD68 (1:100, Cat# ab56297, abcam). The total number of CD31+ cells or CD68+ cells was normalized to the number of DAPI+ (4,6-Diamidino-2-phenylindole, dihydrochlorid) cells in a given field. Sections were cover-slipped with Vectashield with DAPI (Vector Laboratories, Burlingame, CA, USA). Three images were collected from each skeletal muscle section and 3 sections from each animal were imaged and averaged. Total 4–5 biological samples were used for statistical analysis. Digital images were captured at 20× using fluorescent microscope (AxioObserver.Z1; Zeiss, Jena, Germany).

**Statistical analysis:** Results are expressed as mean ± SD (or SEM). Data was processed for statistical analysis with two-way ANOVA; a series of 2 ischemia (control vs. ischemia) × 2 diets (ND vs. HFD); to determine the effect of ischemia and HFD, followed by Bonferroni post-hoc multiple comparisons to assess significant differences between groups (Graphpad-Ver.6). For blood flow data, area under the curve (AUC) across all the time points was calculated. A series of 2 × 2 ANOVAs (gene: WT vs. KO) or 2 diets (ND vs. HFD) with interaction were used to determine the effect of knocking down TXNIP and HFD on body weight, blood glucose and blood flow. A Bonferroni post-hoc multiple comparison test was used for significant interactions. Significance for all tests was determined at alpha = 0.05.

## 3. Results

**Figure 1:**
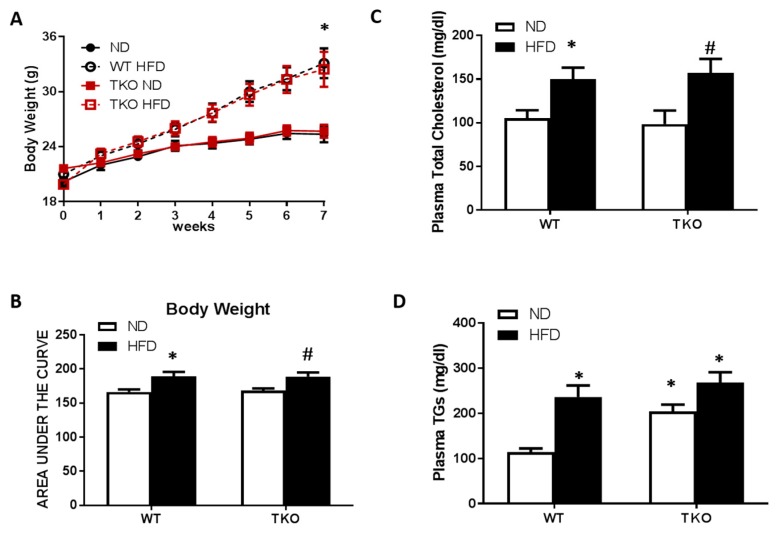
HFD-induced obesity and dyslipidemia in WT and TKO mice. (**A**) Body weight measured weekly for seven weeks in both WT and TKO mice (* *p*-value < 0.05 vs WT-ND or TKO-ND); (**B**) AUC analysis showed an increase in mice weight in WT-HFD and TKO-HFD groups compared to WT-ND and TKO-ND respectively; (*n* = 9–13, * *p*-value < 0.05 vs. WT-ND and # vs. TKO-ND) (**C**) Plasma total cholesterol and (**D**) triglyceride levels showed higher levels in both WT-HFD and TKO-HFD groups, compared to WT-ND. TKO-ND group showed no change in total cholesterol levels, but increased higher basal levels of triglycerides compared with WT-ND (*n* = 8, * *p*-value < 0.05 vs. WT-ND, # vs. TKO-ND). 2 × 2-way ANOVA analysis showed that diet exerted significant effect (*p* < 0.05) on body weight, total cholesterol and triglycerides level and that deletion of TXNIP exerted significant effect (*p* < 0.05) on triglycerides level. HFD: High-fat diet, WT: wild type, TKO: TXNIP knockout, AUC: area under the curve, ND: normal diet, TXNIP: thioredoxin interacting protein.

**HFD causes similar increases in body weight and dyslipidemia in WT and TKO mice.** At 8 weeks of HFD (60% fat), significant increases in weight gain and obesity were observed in both WT-HFD and TKO-HFD compared with their ND-control groups (Figure 1A,B). HFD also caused similar increases in plasma total-cholesterol and triglycerides in WT-HFD and TKO-HFD compared with WT-ND and TKO-ND, respectively. Interestingly, TKO-ND group showed no change in total-cholesterol levels (Figure 1C), but showed a two-fold higher basal level of triglycerides compared with WT-ND (Figure 1D) as part of the phenotype of TKO mice described previously [22].

**Figure 2:**
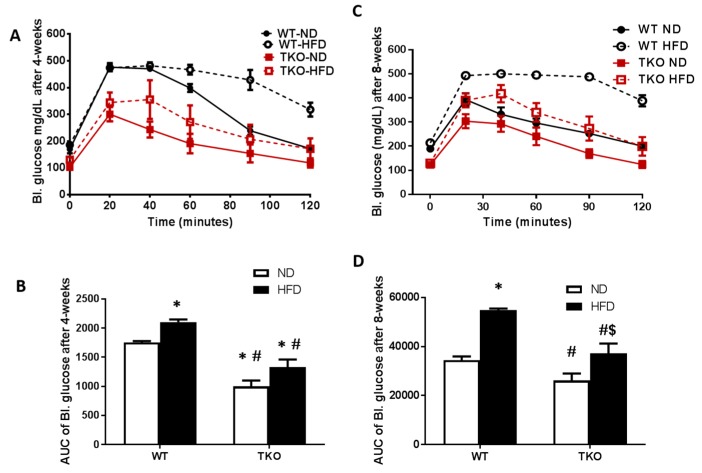
HFD-induced obesity impaired glucose tolerance at 4 weeks and 8 weeks in WT mice but not TKO. IPGTT was performed where peripheral blood glucose levels recorded over time after an IP bolus dose of 2 grams of glucose/kg mouse body weight (zero) and up to 120 min. (**A**) Blood glucose levels (mg/dL) are shown from various groups after 4 weeks of HFD; (**B**) AUC analysis showed that after 4 weeks, HFD significantly impaired peripheral blood glucose response in the WT-HFD group but no significant effect on TKO-HFD. TKO mice showed lower blood glucose level in both ND and HFD groups that were significant compared to WT-ND (*n* = 8, * *p* < 0.05 vs. WT-ND, # *p* < 0.05 vs. WT-HFD). (**C**) Blood glucose levels (mg/dL) are shown from various groups after 8 weeks of HFD; (**D**) AUC analysis showed that HFD impaired peripheral blood glucose response in WT-HFD group and TKO-HFD groups compared to WT-ND and TKO-ND groups, respectively. TKO mice showed lower blood glucose level compared to WT-mice that were significant compared to WT-HFD (*n* = 11, * *p* < 0.05 vs. WT-ND, $ *p* < 0.05 vs. TKO-ND, # *p* < 0.05 vs. WT-HFD). 2 × 2-way ANOVA analysis showed that both diet and TXNIP gene deletion exerted significant effect (*p* < 0.05) on glucose intolerance. IPGTT: Intra peritoneal glucose tolerance test.

**HFD caused early glucose intolerance that was sustained for 8 weeks in WT-mice.** Impaired glucose tolerance and insulin resistance were assessed by the area under the curve (AUC) of blood glucose level following IPGTT in all groups. As shown in Figure 2A,B, WT-HFD caused glucose intolerance evident by significant increase in AUC of blood glucose level after 4 weeks that remained significantly higher after 8 weeks of HFD compared to WT-ND (Figure 2C,D). AUC of TKO mice at baseline (TKO-ND) showed significant reduction compared to WT-ND, suggesting higher insulin sensitivity and better glucose tolerance as previously described [22]. HFD affected TKO mice albeit to a different degree, as AUC from TKO-HFD group showed no difference compared to TKO-ND at 4 weeks, suggesting similar insulin sensitivity (Figure 2A,B). After 8 weeks of HFD, there was mild but significant increase in AUC from TKO-HFD compared to TKO-ND, suggesting impaired insulin and glucose intolerance (Figure 2C,D).

**Figure 3:**
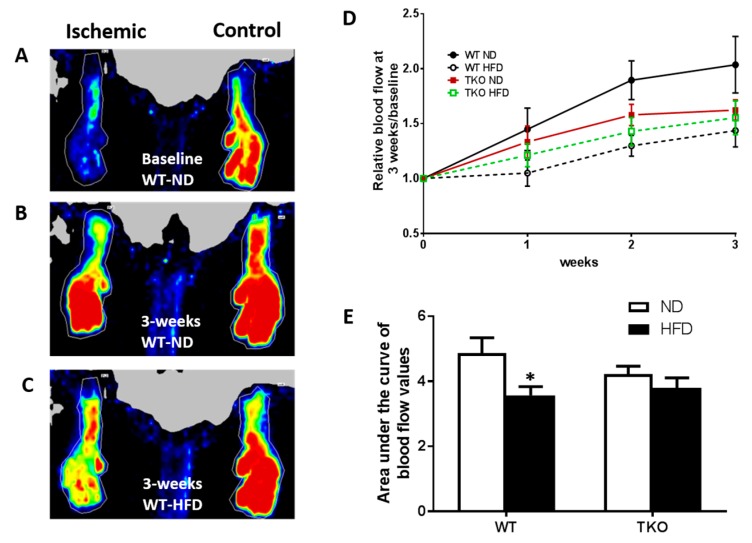
HFD significantly impaired post-ischemic vascular recovery in WT but not in TKO mice. (**A**) Representative image of blood flow of both limbs from WT-ND immediately following unilateral hindlimb ischemia (zero time-point); (**B**) Representative image of blood flow of both limbs from WT-ND 3 weeks post unilateral hindlimb ischemia; (**C**) Representative image of blood flow of both limbs from WT-HFD 3 weeks post unilateral hindlimb ischemia; (**D**) Relative Blood flow measured at baseline and monitored weekly after hindlimb ischemia from various groups; (**E**) AUC analysis showed that HFD significantly reduced blood flow in WT-HFD compared to WT-ND. In contrast, AUC levels from TKO-HFD were not significant from TKO-ND (*n* = 9–12, * *p*-value < 0.05 vs. WT-ND). 2 × 2-way ANOVA analysis showed diet exerted significant effect (*p* < 0.05) on vascular recovery.

**Figure 4:**
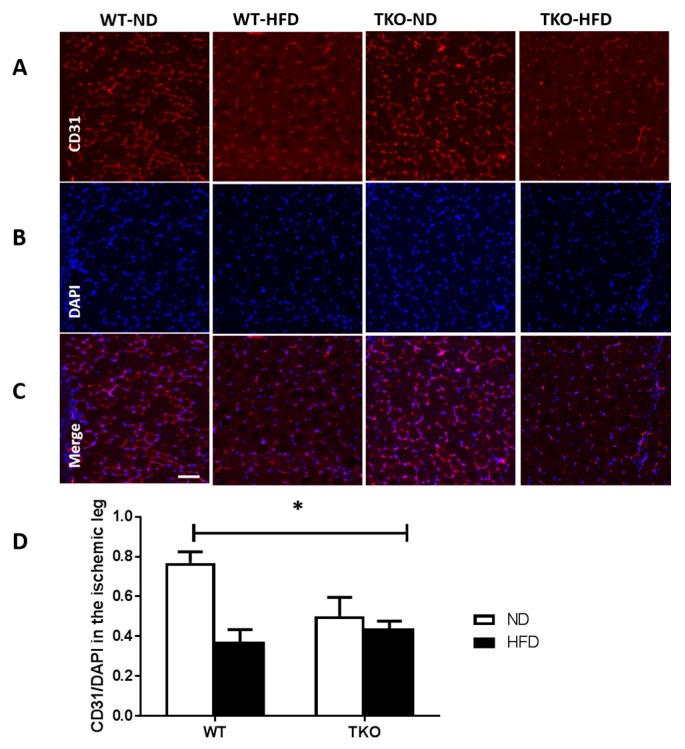
HFD significantly impaired post-ischemic angiogenesis and vascular density in WT but not in TKO. (**A**–**C**) Representative images of skeletal muscles from ischemic side of various groups stained for CD31 (red), DAPI (blue) and Merge CD31 and DAPI (purple); (**D**) 2 × 2-way ANOVA statistical analysis showed a significant interaction among the groups, in which HFD significantly reduced CD31 count in WT-HFD compared to WT-ND. There was no change in CD31 count in TKO-HFD compared to TKO-ND. TKO mice showed lower CD31 count level in both ND and HFD groups that were significant compared to WT-ND (*n* = 6, * *p* < 0.05 gene diet interaction). DAPI: 4,6-Diamidino-2-phenylindole, dihydrochlorid

**HFD significantly impaired angiogenesis, recovery and vascular density in WT but not TKO.** To investigate the role of TXNIP in HFD-altered angiogenic response following ischemia, mice were fed HFD (60% fat) for 4 weeks, a time point of established insulin resistance, then were subjected to unilateral femoral artery ligation model. Angiogenic response and vascular recovery were assessed by measuring blood flow using laser Doppler for up to 3 weeks post hind limb ischemia induction. Data is represented as relative values of ischemic leg (left leg, LL) to non-ischemic side (right leg, RL) and further normalized to baseline readings, recorded immediately after surgery (Figure 3A,C). Interestingly, FIJI analysis of vascular branching density showed that there was no significant difference between sections of gastrocnemius muscles from TKO and WT (Supplementary Figure S1A). This observation came in agreement with our prior findings that retinal vascular density was comparable between WT and TKO mice [23]. Furthermore, there was no significant difference in basal blood flow between WT and TKO mice under both ND and HFD (Supplementary Figure S1B). As shown in Figure 3D,E, HFD significantly impaired vascular recovery in WT-HFD compared to WT-ND at 2 and 3 weeks post-ischemic injury. TXNIP deletion mitigated HFD-impaired angiogenic response, as blood flow was similar among the ND and HFD TKO mice (Figure 3D,E). Next, vascular density and angiogenic response were assessed by staining of the gastrocnemius muscle from the ischemic leg with CD31, the vascular endothelial marker and normalizing its count with DAPI, a nuclear marker (Figure 4A,C). A 2 × 2-way statistical analysis showed a significant reduction in vascular density and impaired angiogenic response in WT-HFD compared to ND (Figure 4D). Interestingly, TKO-ND mice exhibited lower basal blood flow and less vascular density indicated by a lower number of CD31+ cells compared to WT-ND (Figure 3D and Figure 4D). However, HFD did not trigger a further reduction in CD31 count in TKO-HFD compared to TKO-ND. These findings suggest that HFD-enhanced TXNIP expression promotes angiogenic impairment in response to ischemic injury.

**Figure 5:**
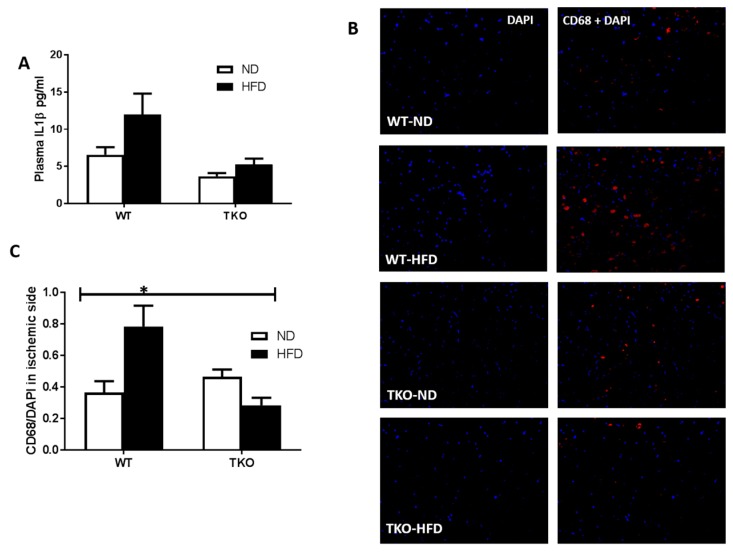
HFD triggered systemic inflammation and significantly increased post-ischemic infiltration of CD68+ cells in WT but not TKO. (**A**) Levels of plasma IL-1β were measured using ELISA and statistical analysis showed a strong trend of HFD to increase levels of plasma IL-1β in WT-HFD but it did not reach significance compared to WT-ND (*n* = 17–24, *p* = 0.059). 2 × 2-way ANOVA statistical analysis showed that TXNIP gene deletion exerted significant effect (*p* < 0.05) on plasma IL-1β level; (**B**) Representative images of skeletal muscles from the ischemic side from WT-HFD stained for CD68+ cells (red), and DAP (blue) from all groups; (**C**) 2 × 2-way ANOVA statistical analysis showed a significant interaction among the groups. HFD-induced CD68+ cells infiltration after ischemia in WT-HFD compared to ND-WT, TXNIP deletion abolished this effect. (*n* = 4–6, * *p* < 0.05 gene diet interaction).

**HFD triggered systemic inflammation and infiltration of CD68+ cells in WT, but not in TKO.** Inflammation is an essential part of the healing process; however, excessive or sustained inflammation can be detrimental to angiogenesis. As shown in Figure 5A, HDF triggered an increase (1.8-fold) in circulating plasma IL-1β levels in WT-HFD group compared to WT-ND group; however, it did not reach statistical significance (*p* = 0.059). A 2 × 2-way ANOVA statistical analysis showed a significant effect of TXNIP deletion where TKO showed significant lower levels than WT-mice (*p* < 0.05) (Figure 5A). Next, in order to assess inflammatory cells infiltration to the site of ischemic injury, the total number of macrophages (CD68+, red) was counted in ischemic sides and normalized to nuclear marker DAPI (blue) from various groups. As shown on Figure 5B,C, HFD-induced obesity led to recruitment of CD68+ cells following ischemia in WT-HFD compared to WT-ND. Nevertheless, deleting TXNIP abolished HFD-mediated macrophage infiltration after limb ischemia (Figure 5B,C). The basal level of CD68+ cells in the non-ischemic leg are shown in Supplementary Figure S2 where HFD exerted a strong trend to increase infiltration of CD68+ Cells compared to ND in WT mice, yet the increase did not reach statistical significance. On the other hand, HFD failed to increase infiltration of CD68+ cells in TKO mice. These results support the potential role of HFD to drive inflammation and the role of TXNIP in abolishing that HFD-mediated inflammation. Deletion of TXNIP did not affect basal inflammatory level where the number of CD68+ cells was not significant between WT-ND and TKO-ND groups. These results support the potential role of HFD to drive inflammation and the role of TXNIP in abolishing that HFD-mediated inflammation.

**Figure 6:**
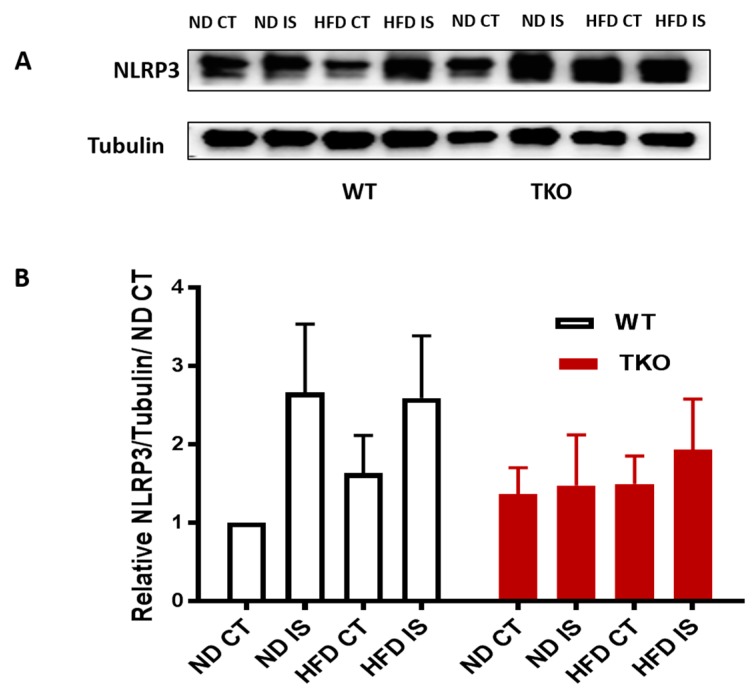
Ischemia increased NLRP3 expression in WT but not in TKO. (**A**) Representative images of Western blots of NLRP3 and Tubulin in gastrocnemius muscles isolated from WT and TKO mice; (**B**) 2 × 2-way ANOVA statistical analysis showed a strong trend of increase in NLRP3 expression following ischemia in WT compared to control (*n* = 4–5, *p* = 0.06 vs. ND CT). HFD tended to increase NLRP3 expression in WT-HFD compared to WT-ND. 2 × 2-way ANOVA statistical analysis showed lack of gene effect where deleting TXNIP did not alter NLRP3 expression following ischemia or HFD compared to WT-ND CT or TKO-ND CT (*n* = 5).

**Figure 7:**
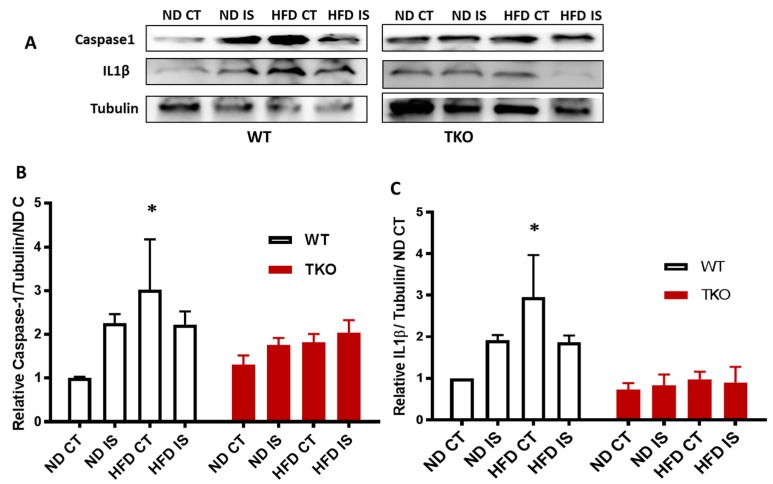
TXNIP deletion prevented HFD-induced NLRP3-inflammasome activation in skeletal muscles; (**A**) Representative images of Western blots of cleaved caspase-1, cleaved IL-1β and Tubulin in gastrocnemius muscles isolated from WT and TKO mice; (**B**) 2 × 2-way ANOVA statistical analysis showed that HFD increased activation of NLRP3 inflammasome evident by significant increases in cleaved caspase-1 in WT-HFD compared to WT-ND but not in TKO mice compared to WT-ND or TKO-ND (*n* = 3–5, * *p* < 0.05 vs. ND CT); (**C**) 2 × 2-way ANOVA statistical analysis showed that HFD increased cleaved IL-1β in WT-HFD compared to WT-ND CT but not in TKO mice compared to WT-ND CT or TKO-ND CT (*n* = 3–5, * *p* < 0.05 vs. ND CT).

**HFD triggers TXNIP-NLRP3-inflammasome activation in WT but not in TKO. ** Next, we examined the impact of HFD and deleting TXNIP on NLRP3-inflammasome in ischemic muscles under ND and HFD conditions. As shown in Figure 6A,B, ischemia tended to increase NLRP3 inflammasome receptor expression ( *p* = 0.06) compared to non-ischemic side in both WT-ND and WT-HFD groups (Figure 6A,B). In TKO mice, there was no significant statistical difference in NLRP3 expression in response to HFD or ischemia compared to WT-ND or TKO-ND (Figure 6A,B). While HFD showed a modest increase in expression of NLRP3 receptor, HFD triggered NLRP3-inflammasome activation evidenced by significant increases in cleaved caspase-1 (Figure 7A,B) and cleaved IL-1β (Figure 7A,C) expressions in WT-HFD compared to WT-ND in the non-ischemic control. In WT, ischemia tended to increase NLRP3 inflammasome activation but it did not reach statistical significance. Interestingly, two-way ANOVA showed significant gene effect, where deleting TXNIP prevented the upregulation of NLRP3-inflammasome activation evident by lack of increase in cleaved caspase-1 (Figure 7A,C) or IL-1β under HFD and ischemic conditions compared to WT-ND or TKO-ND.

**Figure 8:**
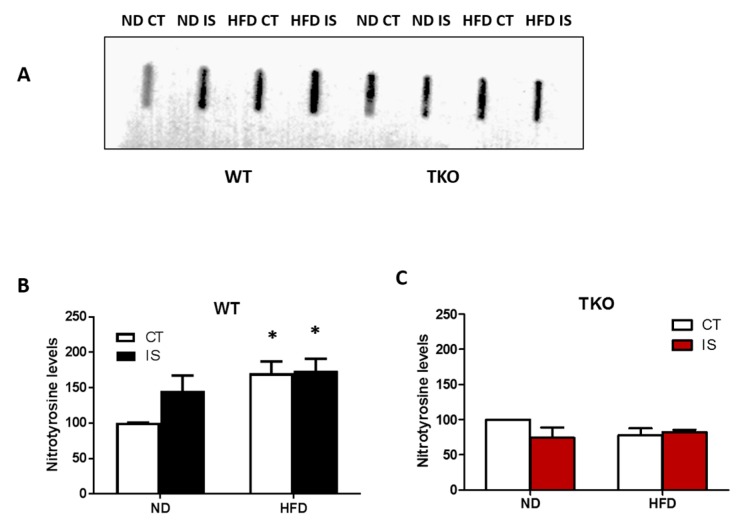
TXNIP deletion prevented ischemia/HFD-induced oxidative stress in skeletal muscles. (**A**) Representative images of slot blots of nitrotyrosine levels in gastrocnemius muscles isolated from WT and TKO, respectively, were extracted from the same gel and same exposure; (**B**) 2 × 2-way ANOVA statistical analysis showed that HFD from both control (CT) and ischemic (IS) muscles significantly increased nitration in WT-HFD compared to WT-ND (*n* = 4–5, * *p* < 0.05 vs. ND CT). Overall, diet exerted significant effect but ischemia showed a trend of increase in nitration compared to control in WT mice but it did not reach statistical significance (*n* = 4). (**C**) Deleting TXNIP abolished the increase in nitration following ischemia or HFD compared to TKO-ND (*n* = 4).

**Deletion of TXNIP abolished HFD- and ischemia-mediated nitrative stress in skeletal muscles.** HFD led to a significant increase in nitrotyrosine level in WT-HFD in both ischemic and non-ischemic side compared to WT-ND control side. Nitrotyrosine levels showed a trend of increase (1.4-fold) in their expression after ischemia alone in WT-ND mice. Interestingly, we found that superimposing an ischemic insult together with HFD did not cause a further increase in tyrosine nitration compared with WT-ND (Figure 8A,B). Deletion of TXNIP prevented HFD- and ischemia-induced nitrotyrosine levels compared to TKO-ND (Figure 8C).

**Figure 9:**
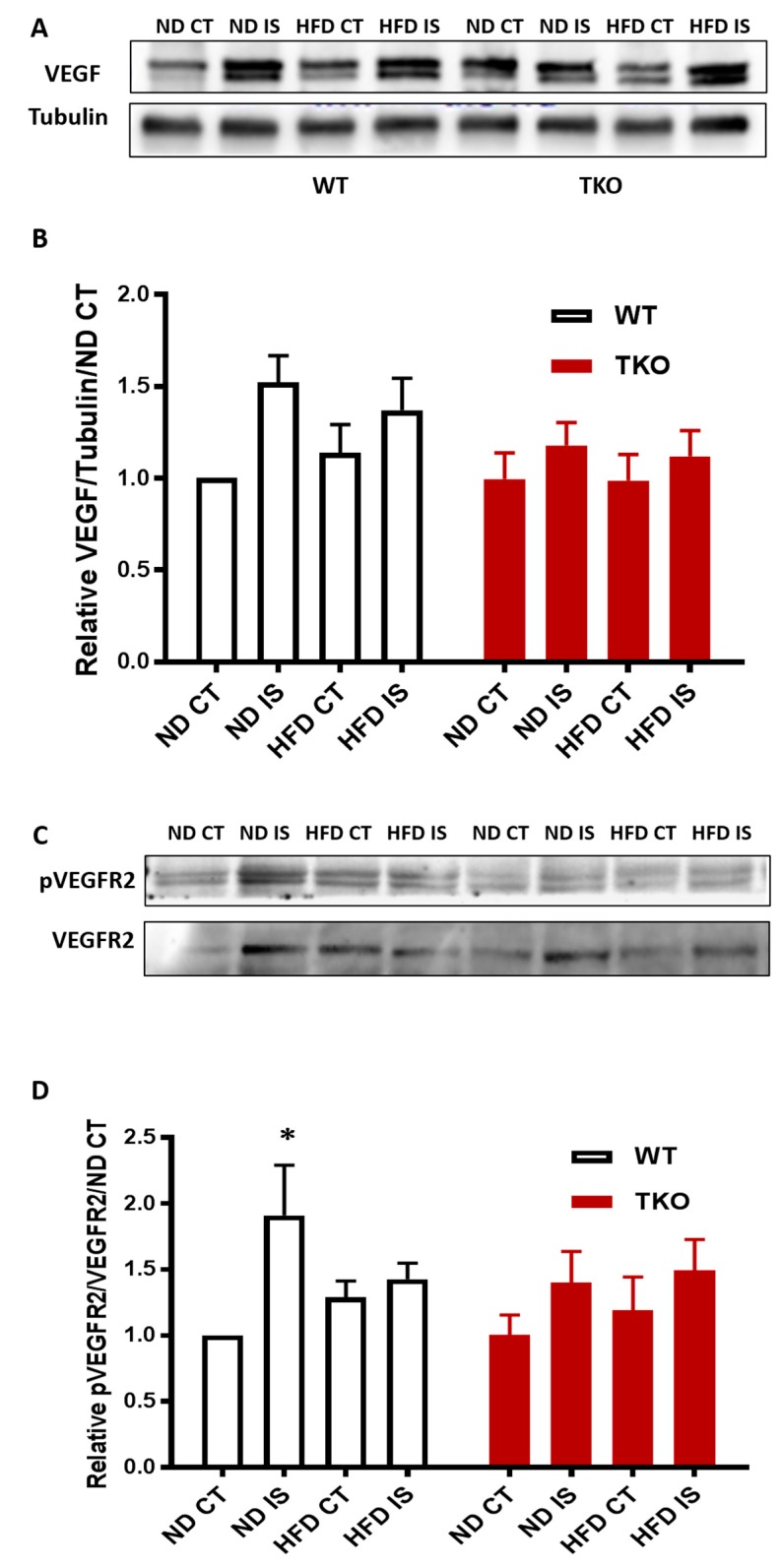
HFD impaired post-ischemic VEGFR2 activation but not levels of VEGF in WT mice. (**A**) Representative images of Western blots of VEGF and Tubulin in gastrocnemius muscles isolated from WT and TKO mice; (**B**) 2 × 2-way ANOVA statistical analysis showed a trend of increase in VEGF expression following ischemia in WT and TKO compared to control but it did not reach statistical significance (*n* = 7); (**C**) Representative images of Western blots of pVEGFR2 and VEGFR2 in gastrocnemius muscles isolated from WT and TKO mice; (**D**) 2 × 2-way ANOVA statistical analysis showed a significant increase in phosphorylation of VEGFR2 following ischemia in WT-ND that was mitigated in the HFD group compared to non-ischemic control (*n* = 5–6, * *p* < 0.05 vs. ND CT). 2 × 2-way ANOVA statistical analysis showed lack of gene effect where deleting TXNIP had minimal effect of altering pVEGFR2 in response to ischemia or HFD compared to WT-ND or TKO-ND (*n* = 6).

**HFD impaired VEGFR2 activation but not levels of VEGF following hind-limb ischemia in WT mice.** Next, we investigated the impact of HFD on VEGF levels and its receptor VEGFR2 angiogenic signal. In response to ischemia, VEGF expression showed a slight increase that was not significant compared to the non-ischemic side in all groups (Figure 9A,B). In WT-ND, ischemia triggered significant VEGFR2 activation compared to the non-ischemic control side compared to WT-ND (Figure 9C,D). However, this therapeutic ischemia-triggered angiogenic signal was impaired by HFD, indicated by the loss of VEGFR2 activation in WT-HFD mice (Figure 9C,D). TKO showed similar levels of VEGF expression in response to HFD or ischemia (Figure 9B). TKO mice showed lower basal level of VEGFR2 activation compared to WT similar to prior observation using retinal angiogenesis model [23]. Interestingly, exposure to HFD or ischemia did not alter VEGFR2 activation in TKO mice compared to TKO-ND (Figure 9D).

## 4. Discussion

Critical limb ischemia (CLI) is a devastating complication of peripheral arterial disease that is closely associated with diabetes. Nevertheless, components of metabolic syndrome including obesity, insulin resistance, dyslipidemia or high blood pressure are established risk factors for CLI (reviewed in [4,24]). A recent report showed that 36% of adults and 17% of youth are obese in the United States [25]. Consumption of high caloric diet is the main drive for the alarming increases in obesity. Here, we tested the hypothesis that high-fat diet (HFD)-induced obesity will impair ischemia-induced angiogenesis and vascular recovery via TXNIP-NLRP3 inflammasome activation. The main findings of our study are: (1) HFD-induced obesity resulted in impaired glucose tolerance and dyslipidemia in both WT and TKO; (2) Deleting TXNIP prevented HFD-impaired vascular recovery following hind-limb ischemia; (3) HFD triggered systemic and local infiltration of CD68+ macrophages to ischemic limb in WT but not TKO; (4) TXNIP deletion prevented HFD-mediated inflammasome activation and oxidative stress; (5) HFD did not affect VEGF level but impaired VEGFR2 activation in WT following hind-limb ischemia. We believe that this is the first report that demonstrates the impact and elucidates the mechanisms by which HFD impairs angiogenic response and vascular recovery in response to ischemic insult. Early intervention with treatments that target TXNIP-NLRP3 inflammasome activation will provide effective therapeutics for obese subjects presenting with CLI.

We observed that HFD-induced obesity impaired blood flow recovery and reduced vascular density after ischemia in WT but not in TKO. Similarly, silencing TXNIP in diabetic mice restored blood flow recovery and capillary density following hind-limb ischemia [16]. The protective effects of global TXNIP deletion can be attributed to a plethora of pathways that are regulated by TXNIP. For instance, TXNIP regulates the cellular oxidative-reductive state through inhibiting thioredoxin, one of the main antioxidant systems in the body. TXNIP inhibits the reducing ability of thioredoxin via binding to the catalytic active sites of the enzyme (reviewed in [26]). In addition, TXNIP is known to be one of the master regulators of metabolism, a well-established inhibitor of glucose uptake, mediator of insulin resistance, and is primarily involved in proper fatty acid utilization [27,28,29]. Our results have indicated that TKO mice are more insulin-sensitive and consistently had lower fasting blood glucose levels despite the comparable weight gain after HFD. Their metabolic profiling also showed comparable plasma cholesterol and triglycerides levels after HFD, but a higher basal level of plasma triglycerides in TKO-ND. These observations are in line with previously published reports that attributed higher basal levels of plasma triglycerides due to the altered ratio of NADH to NAD^+^ which down-regulates the citric-acid cycle, sparing fatty acids for triglyceride and ketone body production [22,30]. Our results showing that TXNIP deletion preserves insulin sensitivity without affecting weight gain or obesity lend further support to prior studies using TKO in HFD diet-induced and genetic obesity models [31,32].

Clinically, insulin resistance has been shown to result in peripheral arterial disease. A recent study showed that the higher the HOMA-IR (homeostatic model assessment of insulin resistance) value becomes, the more the prevalence of peripheral arterial disease increases to a clinically significant extent [33]. Experimentally, Kubota et al. reported that impaired insulin signaling in endothelial cells, due to reduced insulin receptor substrate 2 expression and insulin-induced phosphorylation of endothelial nitric oxide synthase (eNOS), caused attenuation of insulin-induced capillary recruitment and insulin delivery, which in turn reduced glucose uptake by skeletal muscle in HFD mice [34]. Moreover, treatments that restore insulin sensitivity in endothelial cells and hence insulin-induced signaling reverses reduction in vascular recovery (reviewed in [35]). Thus, TXNIP-mediated insulin resistance can contribute to the impaired vascular recovery in response to ischemic insult in WT mice. Findings from type 1 or type 2 diabetic mice that underwent limb ischemia showed significant increases in nitrotyrosine levels that coincided with decreases in eNOS expression in the gastrocnemius muscle [36]. Interestingly, vascular recovery was further impaired in type 2 diabetic mice more than type 1 diabetic mice due to extensive fatty infiltration [36]. Although we did not directly assess eNOS expression or nitric oxide, we measured levels of nitrotyrosine as surrogate marker of peroxynitrite, the combination product of nitric oxide and superoxide anion. We found that HFD alone increased nitrative stress with or without ischemia, which was mitigated by TXNIP deletion. These results are in agreement with prior studies showing that ischemia and HFD increase oxidative and nitrative stress in retina [12], in myocardial ischemia [37] and brains from HFD mice after ischemic stroke compared to non-ischemic controls [38]. We did not see additive effects of HFD and ischemia in skeletal muscles that might be attributed to the sensitivity of the method used to detect nitrotyrosine levels.

In response to various disorders, increased reactive oxygen species enhances the dissociation of TXNIP from thioredoxin. This effect results in increasing TXNIP availability to interact with NLRP3 leading to inflammasome activation (reviewed in [9]). Activated NLRP3 oligomerizes with the apoptosis-associated speck-like adaptor protein forming caspase-1 activating complex known as NLRP3 inflammasome. Activated caspase-1 enzyme in turn cleaves premature pro-inflammatory cytokines including IL-1β and IL-18 into their mature form causing inflammation [10,11]. Although inflammatory response is necessary for healing, excess inflammatory reaction can lead to detrimental effect. Therefore, it is pretty challenging to find optimum therapeutic strategy. We and others have shown the link between TXNIP-NLRP3 interaction and inflammasome activation [12,14,39,40]. Nevertheless, whether the contribution of TXNIP-NLRP3 inflammasome to impaired vascular recovery in ischemic limb remains unexplored, in the current study, we investigated whether TXNIP-mediated inflammation contributes to HFD-impaired vascular recovery following hind-limb ischemia. We found that HFD led to systemic and local inflammation evident by increased plasma IL-1β levels and CD68+ cells (macrophage) infiltration in ischemic muscle. Our results lend further support to prior studies showing that HFD increased infiltration of CD68+ cells [41,42] and that HFD-mediated NLRP3-inflammasome activation resulted in elevated systemic IL-1β levels [43,44]. These effects were abolished by deleting TXNIP (Figure 5). Moreover, we found that HFD upregulated caspase-1 and IL-1β expression, indicating NLRP3 inflammasome activation. Interestingly, superimposing HFD with ischemic insult did not result in further increase in NLRP3 and inflammatory cytokines expression. We found also that ischemia led to upregulation of NLRP3 expression and tended to increase caspase-1 and IL-1β expression under ND. Finally, we demonstrated that TXNIP deletion restored NLRP3, caspase-1 and IL-1β expression and abolished HFD/ischemia-mediated inflammation (Figure 6 and Figure 7). These results suggest that inhibiting TXNIP can target the harmful inflammatory response without impairing the recovering process. In agreement, several recent studies demonstrated the protective effects of targeting TXNIP-NLRP3 inflammasome in various disorders [45,46,47].

VEGF via binding to VEGFR2 plays a crucial role in enhancing angiogenic-like response in endothelial cells including migration and proliferation (reviewed in [15]). Furthermore, several experimental studies showed the advantage of the administration of recombinant VEGF in inducing reparative angiogenesis in response to hind-limb ischemia [48,49]. Recently, Dunn et al. have demonstrated a reduction in VEGF expression in ischemic skeletal muscles isolated from diabetic mice compared to non-diabetic group after hind-limb ischemia. In the current study, we found that ischemia tended to increase VEGF expression compared to the non-ischemic side in WT mice under ND or HFD, but it did not reach significance. Deletion of TXNIP had a modest effect on VEGF levels in response to ischemia or HFD. The interplay between thioredoxin system including thioredoxin, TXNIP and VEGF expression is well documented in the literature (reviewed in [17]). While one study showed that enhanced TXNIP expression stimulates VEGF expression [18], others showed that enhanced TXNIP can downregulate VEGF production [16,19,20]. We and others have demonstrated that TXNIP is required for VEGF/VEGFR2 angiogenic signal but it does not affect VEGF levels [23,50,51,52]. Indeed, HFD abolished ischemia-induced VEGFR2 activation seen in WT-ND. Of note, these results were obtained using whole muscle lysate, suggesting that altered activity of VEGFR2 receptor can be due to impaired angiogenic signal in endothelial cells and/or involvement of other cells such as dying myofibers, and proliferating muscle stem cells. It is interesting that both diabetes and HFD-obesity-mediated impairment of angiogenesis is mainly through altering of VEGF production and VEGFR2 activation, respectively. Similar to our previous observation [23], deletion of TXNIP was associated with lower basal VEGFR2 activation, an effect that was not changed in response to HFD or ischemia.

In summary, the current pharmacotherapy intervention for CLI is limited, resulting in limb amputation in many cases. Our findings demonstrate that HFD-induced obesity can compromise vascular recovery in response to ischemic insult. Our results showed clearly that HFD stimulates TXNIP-NLRP3 inflammasome, IL-1β as well as infiltration of CD68+, which in combination with ischemic insult exerted minimal additional effects. In contrast, ischemia had a more pronounced effect on blood flow, vascular density and VEGF/VEGFR2. Deletion of TXNIP restored blood flow, reduced nitrative stress and blunted HFD-inflammasome-mediated inflammation. Our results also show that, while TKO and WT show comparable basal level of blood flow and vascular density, upon ischemic insult, TKO show less angiogenic response under normal condition, which, in contrast to WT-HFD mice, did not deteriorate under HFD condition. Targeting TXNIP-NLRP3 inflammasome can provide potential therapeutic target in obesity-induced vascular complication.

## Figures and Tables

**Table 1:** A summary of sources of antibodies used to detect protein expression using Western Blot.

Antibodies	Catalog Number	Company
anti-VEGF	Cat.# 676472 Cat.# ABS82	Abcam Millipore
anti-pVEGF receptor-2 (Tyr996)	Cat.#2474	Cell Signaling
anti-VEGF receptor 2	Cat.#2472	
anti-GAPDH	Cat.# 5174	
anti-IL-1β	Cat.# ab9722	Abcam
anti-tubulin	Cat.# ab4074	
anti-NLRP-3	Cat.# LS-B4321	LifeSpan Biosciences
anti-nitrotyrosine	Cat.# 05-2333	Millipore

VEGF: vascular endothelial growth factor; GAPDH: Glyceraldehyde 3-phosphate dehydrogenase; NLRP3: nucleotide-binding oligomerization domain-like receptor protein 3.

## Supplementary Materials

The following are available online at www.mdpi.com/2076-3921/6/3/47/s1, **Figure S1.** (**A**). Representative images and statistical analysis show that there was no significant difference in % vascular branching density assessed by FIJI analysis of isolectin-stained sections of gastrocnemius muscles from TKO-ND and WT-ND (*n* = 4–5); (**B**). Vascular recovery was assessed by measuring blood flow using laser Doppler of ischemic leg (left leg, LL) compared to the non-ischemic side (right leg, RL) control in each animal at base. There was no significant difference in basal blood flow between WT and TKO mice under both ND and HFD. **Figure S2.** (**A**). Representative images of skeletal muscles from the control non-ischemic side from various groups stained for CD68+ cells (red), and DAP (blue). (**B**). 2 × 2-way ANOVA statistical analysis showed no significant interaction among the groups. HFD caused a strong trend to increase infiltration of CD68+ cells in WT-HFD compared to ND-WT, TXNIP deletion abolished this effect. (*n* = 4–5).
