# Supplementary material for: Deletion of TXNIP Mitigates High-Fat Diet-Impaired Angiogenesis and Prevents Inflammation in a Mouse Model of Critical Limb Ischemia
Source: Antioxidants (Basel). 2017 Jun 29;6(3):47. doi: 10.3390/antiox6030047 (PMC5618075; doi:10.3390/antiox6030047)
Supplement: Supplementary file 1 [file antioxidants-06-00047-s001.pdf]

antioxidants

MDPI

Supplementary Materials

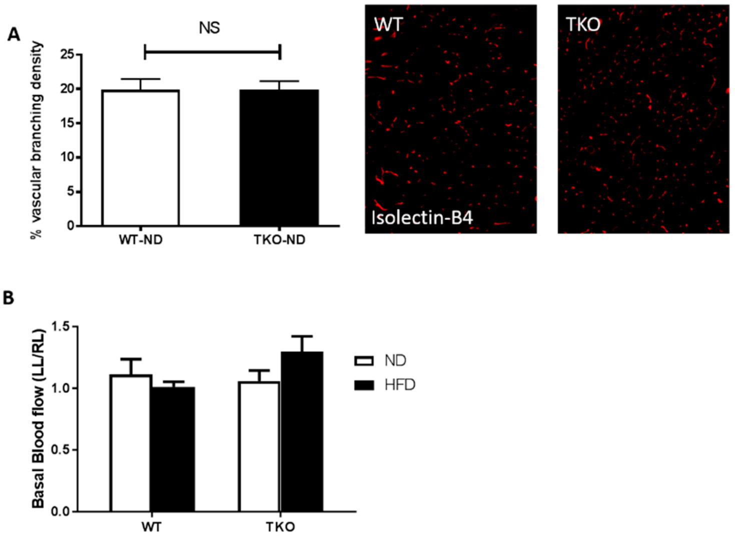

Figure S1. (**A**). Representative images and statistical analysis show that there was no significant difference in % vascular branching density assessed by FIJI analysis of isolectin-stained sections of gastrocnemius muscles from TKO-ND and WT-ND (n = 4–5); (**B**). Vascular recovery was assessed by measuring blood flow using laser Doppler of ischemic leg (left leg, LL) compared to non-ischemic side (right leg, RL) control in each animal at base. There was no significant difference in basal blood flow between WT and TKO mice under both ND and HFD.

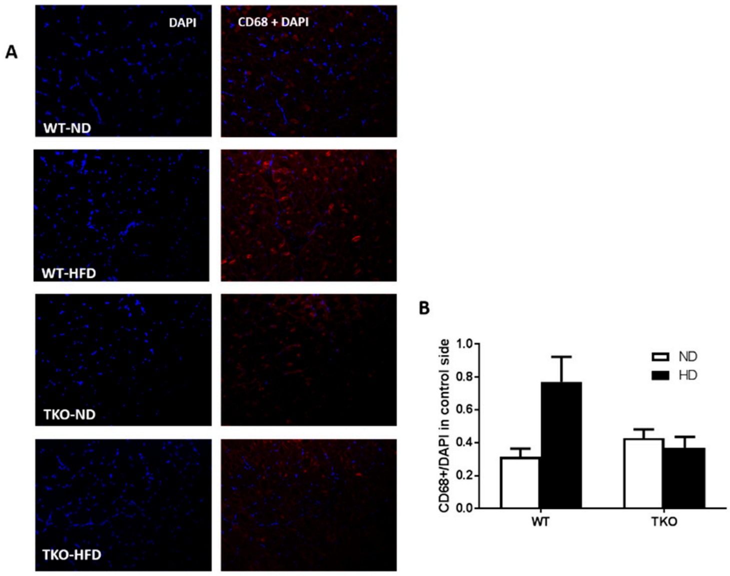

Figure S2. (**A**). Representative images of skeletal muscles from control non-ischemic side from various groups stained for CD68+ cells (red), and DAP (blue). (**B**). 2 × 2-way ANOVA statistical analysis showed no significant interaction among the groups. HFD caused a strong trend to increase infiltration of CD68+ cells in WT-HFD compared to ND-WT, TXNIP deletion abolished this effect. (n = 4–5).
